# Basalt/Wood Hybrid Composites Based on Polypropylene: Morphology, Processing Properties, and Mechanical and Thermal Expansion Performance

**DOI:** 10.3390/ma12162557

**Published:** 2019-08-11

**Authors:** Anna Kufel, Stanisław Kuciel

**Affiliations:** Faculty of Mechanical Engineering, Cracow University of Technology, ul. Warszawska 24, 31-155 Cracow, Poland

**Keywords:** basalt fiber, wood–plastic composites, thermoplastic, thermal expansion

## Abstract

The main aim of this study was to investigate the effect of basalt fiber (BF) reinforcement in wood–plastic composites (WPCs). Basalt/wood hybrid composites based on polypropylene (PP) were prepared with different percentages of the reinforcement (the total fiber content was 10 wt%, 15 wt%, and 20 wt%). The BCS17-6.4-KV16 chopped basalt fibers with nominal diameter of 17 μm, cutting length of 6.4 mm, and wood fibers—Lignocel C 120 with the particle size of 70–150 µm—were used as a reinforcement. Composites were produced by the injection molding method. The density of the produced composites and their processing properties such as Vicat softening point and shrinkage were determined. In addition, the thermal expansion behavior of filled plastic composites was investigated. Mechanical tests were subsequently performed to evaluate the tensile, flexural, and impact properties at various temperatures (i.e., at −24 °C, 23 °C, and 80 °C) and after soaking in water. Scanning electron microscopy images were acquired to assess the effects of reinforcement and homogenization of mixtures and to determine the characteristics of the microstructure. The results showed that the hybridization process improved the tensile and flexural properties of reinforced wood composites. Moreover, the incorporation of high-strength basalt fibers into the composite led to increased stiffness. Even a small addition of 10 wt% total fibers led to a significant decrease in shrinkage and coefficient of thermal expansion.

## 1. Introduction

In recent years, there has been growing attention in the need to protect ecological and environmental resources. Wood–plastic composites (WPCs) contain wood, plastics, and additives and are manufactured by use of thermoplastic shaping techniques such as extrusion, injection molding, and compression molding. Color and texture of WPCs are comparable to wood; hence, WPCs are widely used in decking, fencing, railing, playground equipment, window and door frames, and automobiles [1]. To improve the mechanical properties of natural fiber-reinforced composites a hybridization with inorganic fillers can be applied [2]. The disadvantages of one component can be eliminated by the addition of another fiber [3,4]. One of the new solutions in the production of composites is adding basalt fibers (BFs) to WPCs. BFs are usually produced by making use of basalt rocks, which are found globally. That is why BFs are considered as natural in spite of they are not biodegradable. Basalt is a natural material found in volcanic rocks originating from frozen lava, with a melting temperature ranging from 1500 °C to 1700 °C [5,6]. BF has low cost and excellent properties such as high-temperature resistance, corrosion resistance, thermal insulation, and sound absorption and low moisture absorption [7].

Many studies have attempted to improve the mechanical properties of WPCs. For example, WPCs could include glass fibers or carbon fibers [8,9]. Al-Maadeed et al. [10] studied the mechanical and thermal properties of recycled polypropylene (PP)/glass fiber/wood flour composites. They found that adding glass fibers to the recycled PP increased modulus, strength, and hardness but decreased ductility, however, adding wood fibers as a second reinforcement had the opposite effects. In another study [11], date palm wood flour/glass fiber reinforced recycled polypropylene composites were investigated. The tensile strength and modulus of elasticity increased even more by further adding glass fibers. Valente et al. [12] studied hybrid composites with wood flour and recycled glass fibers. The addition of recycled glass fibers in hybrid composites improved water absorption behavior compared to that of WPCs. Many studies have indicated that BFs are a good alternative to glass fibers [13,14]. Furthermore, the manufacturing process of BFs is similar to that of glass fibers. BFs have been used within studies as reinforcements in thermoplastic matrices in the last years [15,16]; however, the usage in WPCs as reinforcement was rarely reported. Chen et al. [17] added BFs into a commercial WPC. The improvement in the tensile and flexural strength of WPCs based on high-density polyethylene was affected by adding BFs. In another study [18], the addition of modified basalt fibers to WPCs provided better flexural and impact properties. A vinyl triethoxy silane coupling agent was used to modify the basalt fibers. An indispensable activity to improve the reinforcement of basalt fibers is to suitably treat the surface. Surface modifications of the fibers or by making use of bulk modifications of the polymer matrix can improve the adhesion between matrix and basalt fibers. The necessity of using different compatibilizers, among which maleic anhydride-grafted polypropylene (MAPP) can be mentioned and widely used, is given [19]. MAPP is commonly grafted to the same polymer as that used as the matrix to ensure compatibility between the matrix and the coupling agent. MAPP, produced by grafting MA to PP, can react with the hydroxyl groups on fiber surfaces, leading to covalent or hydrogen bonding. It can be used as an additive during processing or grafted to the fiber before processing. Comparing all the methods which lead to an improvement of interfacial bonding, coupling BFs with MAPP could be regarded as the most successful one [20]. Shrinkage of the products is very important parameter. Shrinkage is a volume change phenomenon; however, it affects the use of the final products. For example, shrinkage will lead to an inaccurate size; hence, it is necessary to reduce PP shrinkage. The shrinkage of tested composites was lower than that for our previously tested basalt/carbon PP composites [21].

This research aimed to develop and characterize novel basalt/wood hybrid composites based on PP produced by the injection molding method. The major objective of the present study was to investigate the impact of adding basalt fibers to WPCs on the thermal and mechanical properties of such hybrid composites. This study also covered the characterization of a fiber-matrix interface by scanning electron microscopy to explain the mechanism of reinforcing hybrid composites. In addition, improvements in processing properties such as softening temperature and thermal stability were monitored. 

## 2. Materials and Methods 

### 2.1. Materials

The matrix material of hybrid composites was PP Moplen HP 500N, which was produced by Basell Orlen Polyolefins (Płock, Poland). It was reinforced by adding basalt and wood fibers in the same percent by weight, i.e., the total fiber content was 10 wt%, 15 wt%, and 20 wt% (Table 1). The BCS17-6.4-KV16 chopped basalt fibers with nominal diameter of 17 μm, cutting length of 6.4 mm, and appropriate sizing for polyolefins were supplied by Basaltex, (Wevelgem, Belgium). Wood fibers—Lignocel C 120 with the particle size of 70–150 µm—were obtained from J. Rettenmaier & Söhne GmbH, (Rosenberg, Germany). The anhydride maleic PP SCONA TPPP 9112 GA (MAPP), supplied by Byk (Altana AG, Wesel, Germany), was used. The standard dumbbell samples were prepared by injection on an Engel ES 200/40 HSL injection molding machine (ENGEL GmbH, Schwertberg, Austria) after compounding fibers and polypropylene on MARIS TM30 co-rotating twin-screw extruder (Maris America Corp., Windsor Mill, MD, USA). The temperatures in the subsequent zones ranged from 180 °C to 220 °C, and the injection speed ranged from 60 to 90 mm/s. The processes of compounding and injection were carried out in the Laboratory of Plastics Technology operating under Grupa Azoty SA in Tarnów, Poland.

### 2.2. Methods of Testing

#### 2.2.1. Determination of True and Theoretical Density

The density of the produced composites was measured using the hydrostatic method at room temperature on a RADWAG WAS 22W scale (Radwag, Radom, Poland), according to EN ISO 1183. The samples were measured in ethanol medium. On the basis of the assumed theoretical weight fraction of the filler and matrix in the tested composites, as well as the density of the fibers and matrix, the theoretical volume fraction of the fillers in the tested composites was calculated. For the tested composites, the volume fraction of basalt fibers was calculated using the following equation:

(1) $$VF_{f1}=\frac{1}{1+\frac{\rho_{f1}}{\rho_{f2}}+\frac{\rho_{f1}}{\rho_{m}}\left(\frac{1}{WF_{f1}}-2\right)}$$

where *ρ*_*f*1_ is density of basalt fibers, *ρ*_*f*2_ is density of wood fibers, *ρ_m_* is density of the matrix, and *WF*_*f*1_ is weight fraction of basalt fibers.

The theoretical density of the tested composites was calculated according to the basic law of mixtures:

(2) $$\rho_{c}=\rho_{f1}VF_{f1}+\rho_{f2}VF_{f2}+\rho_{m}\left(1-VF_{f1}-VF_{f2}\right)$$

A comparison of the theoretical density and true density allowed to evaluate the appropriateness of component dosing and feeding in the process of producing composite granulates by compounding.

#### 2.2.2. Water Absorption

Water absorption was carried out according to ASTM D570-98 standard. The specimens of PP and composites were immersed in distilled water at room temperature and weighed after 1, 14, and 30 days by using an electronic weighing balance (RADWAG WAS 22W). The samples were removed from the water bath, and their weight was measured after surface drying. Water absorption was calculated using the following equation:

(3) $$\%W=\frac{W_{n}-W_{0}}{W_{0}}\times 100$$

where *W*_0_ is the initial weight of the sample, *W_n_* is the weight of the saturated sample, and %*W* is the percentage increase in weight.

#### 2.2.3. Mechanical Testing

Specimens for mechanical testing were conditioned at 23 °C/50% relative humidity for at least 80 h according to ISO 291 for test room conditions. Tensile tests were carried out according to PN-EN ISO 527-1:2012. MTS Criterion Model 43 testing machine (MTS Systems Corp., Eden Prairie, MN, USA) with a maximum load range of up to 30 kN and with a traverse speed of 5 mm/min was used. For the accurate measurement of displacement to allow the determination of the tensile modulus, an MTS 634.31F axial extensometer was used. In addition to the tensile tests, three-point flexural tests were also conducted for the compositions according to PN-EN ISO 178:2011. Machine control was performed using MTS TestSuite TW software 1.0 (MTS Systems Corp.). Charpy impact test was carried out in accordance with PN-EN ISO 179-2 standard using the Zwick/Roell MTS-SP (Zwick Roell Group, Ulm, Germany) testing machine. The average unnotched impact strength was calculated. The mechanical tests were performed at various temperatures of potential applications. The tests were conducted at −24 °C, 23 °C, and 80 °C by using a temperature chamber (Instron, Norwood, USA). To assess the influence of absorbed water, all compositions were tested in a conditioned state and after 30 days of soaking in water. 

#### 2.2.4. Rule of Hybrid Mixtures

By making use of the Rule of Hybrid Mixtures (RoHM) equation [22,23] an evaluation of the elastic modulus of hybrid short fiber composites is possible. Assuming that there is no interaction between the two single systems, it is possible to apply the iso-strain, i.e.,

(4) $$\varepsilon_{c}=\varepsilon_{c}{}_{1}=\varepsilon_{c_{2}}$$

where *ε_c_* is the strains of the hybrid composite, *ε*_*c*1_ is the first system, and *ε*_*c*2_ is the second system. Force equilibrium involves that

(5) $$E_{c}\varepsilon_{c}=E_{c1}\varepsilon_{c1}V_{c1}+E_{c2}\varepsilon_{c2}V_{c2}$$

Then, the modulus of the composite can be estimated from the RoHM equation. The interaction between the two systems can be neglected as follows:

(6) $$E_{c}=E_{c1}V_{c1}+E_{c2}V_{c2}$$

where *E_c_* is the elastic modulus of the hybrid composite, *V*_*c*1_ and *V*_*c*2_ are the relative hybrid volume fraction of the first and second systems, respectively. Please note that the following expressions are valid for the assumed system:

(7) $$V_{c1}+V_{c2}=1$$

(8) $$V_{c1}=V_{f1}/V_{t}$$

(9) $$V_{c2}=V_{f2}/V_{t}$$

(10) $$V_{t}=V_{f1}+V_{f2}$$

where *V_t_* is the total reinforcement volume fraction. In addition, *V*_*f*1_ + *V*_*f*2_ is used as reinforcement volume fraction for the calculation of the elastic modulus (*E*_*c*1_ and *E*_*c*2_) of both the single composites. 

#### 2.2.5. Halpin–Tsai Equation

To calculate the elastic modulus of hybrid composites the modified Halpin–Tsai equation was used. The values of the composites modulus *E*_11_ and *E*_22_ can be derived using the modified Halpin–Tsai model as follows [24]:

(11) $$E_{11}=\left\{\frac{1+2\frac{l_{f1}}{d_{f1}}\eta_{L1}V_{f1}}{1-\eta_{L1}V_{f1}}E_{m}\right\}+\left\{\frac{1+2\frac{l_{f2}}{d_{f2}}\eta_{L2}V_{f1}}{1-\eta_{L2}V_{f2}}E_{m}\right\}$$

(12) $$E_{22}=\left\{\frac{1+2\eta_{T1}V_{f1}}{1-\eta_{L1}V_{f1}}E_{m}\right\}+\left\{\frac{1+2\eta_{T2}V_{f2}}{1-\eta_{T2}V_{f2}}E_{m}\right\}$$

(13) $$\eta_{L1}=\frac{\left(E_{f1}/E_{m}\right)-1}{\left(E_{f1}/E_{m}\right)+2\left(l_{f1}/d_{f1}\right)}$$

(14) $$\eta_{T1}=\frac{\left(E_{f1}/E_{m}\right)-1}{\left(E_{f1}/E_{m}\right)+2}$$

(15) $$\eta_{L1}=\frac{\left(E_{f2}/E_{m}\right)-1}{\left(E_{f2}/E_{m}\right)+2\left(l_{f2}/d_{f2}\right)}$$

(16) $$\eta_{T1}=\frac{\left(E_{f2}/E_{m}\right)-1}{\left(E_{f2}/E_{m}\right)+2}$$

For random distribution:

(17) $$E_{random}=\frac{3}{8}E_{11}+\frac{5}{8}E_{22}$$

where:

*E* is the elastic modulus
*V* is the volume fraction
*l* is length of the fiber
*d* is diameter of the fiber
*m* is matrix
*f*_1_ and *f*_2_ are first fiber and second fiber, respectively.

#### 2.2.6. Vicat Softening Temperature Measurement

The softening temperature was determined by the Vicat method (ISO 75) using a Ceast HDT and Vicat Tester Type 6520 (CEAST, Pianezza, Italy) machine using method A50 with a force of 10 N and a heating rate of 50 °C/h.

#### 2.2.7. Linear Shrinkage

The percentage linear shrinkage (*LS*) of the specimen was calculated as follows:

(18) $$LS=\frac{L_{1}-L_{2}}{L_{1}}\times 100\%$$

where *LS* is linear shrinkage, *L*_1_ is selected dimension in the form [µm], and *L*_2_ is the same dimension measured on the mold at a certain temperature and pressure [µm].

#### 2.2.8. Composite Morphology

SEM structure images were acquired on tensile-test fracture surfaces of specimens using scanning electron microscope JEOL JSN5510LV (JEOL Ltd., Tokyo, Japan). The accelerating voltage was 20 kV. All samples were coated with gold using Cressington 108 auto sputter coater (Cressington Scientific Instruments, Watford, UK) before observation.

#### 2.2.9. Thermal Expansion

The thermal properties of composites were assessed on the NETZSCH 402 F1 Hyperion device (NERZSCH Group, Selb, Germany), in which the samples, with a dimension of 4 mm × 4 mm × 20 mm, were placed vertically. Samples were cut from dumbbell samples, produced by the injection molding method. Five specimens were used for each group. Dilatometric analysis consists of measuring the length of the sample (L) as a function of temperature (T). The length and temperature data were recorded and analyzed with Proteus software. The samples were cooled from 30 °C temperature to −60 °C, heated to 140 °C, and finally cooled to −60 °C temperature. The heating and cooling rates were kept constant at 10 °C/min. The measurement was carried out in an air atmosphere with a flow rate of 60 mL/min, while the measurement below 20 °C was possible due to the use of nitrogen. The coefficient of linear thermal expansion (*α_L_*) was calculated as:

(19) $$\propto_{L}=\frac{1}{L}\frac{dL}{dT},\;1/K$$

where *L* is the linear dimension of the test sample and *dL*/*dT* is the rate of change in the linear dimension per unit temperature.

## 3. Results and Discussion 

### 3.1. Physical and Processing Properties

The properties of tested materials are described in Table 2 with the results of the density measurements compared with theoretical density, shrinkage value, and Vicat softening temperature. There are some discrepancies between the measured density and the calculated one. A slightly lower value of the measured density may indicate a lower fiber content than was assumed. The addition of basalt and wood fibers caused, proportionally to the amount of the fibers, an increase in the density of the obtained composites. As can be seen, basalt fibers and wood fibers can reduce shrinkage of PP effectively. Composites with 10 wt% of total fibers had lower shrinkage than composites with 10 wt% glass fibers tested by Wu et al. [25]. Vicat softening temperature was enhanced for all composites. Adding as much as 10 wt% of total fibers increased Vicat softening temperature from 150.6 °C to 154.3 °C.

### 3.2. Static Tensile Test

**Figure 1:**
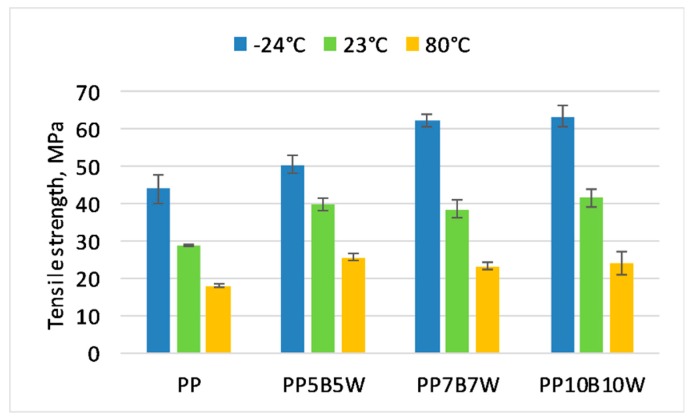
Tensile strength of the tested materials at −24 °C, 23 °C, and 80 °C.

**Figure 2:**
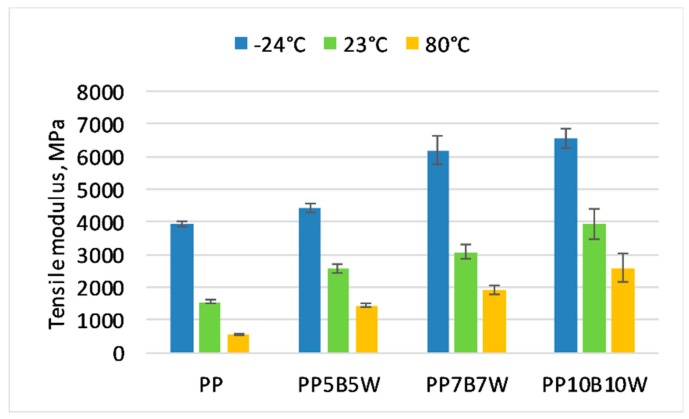
Tensile modulus of the tested materials at −24 °C, 23 °C, and 80 °C.

**Figure 3:**
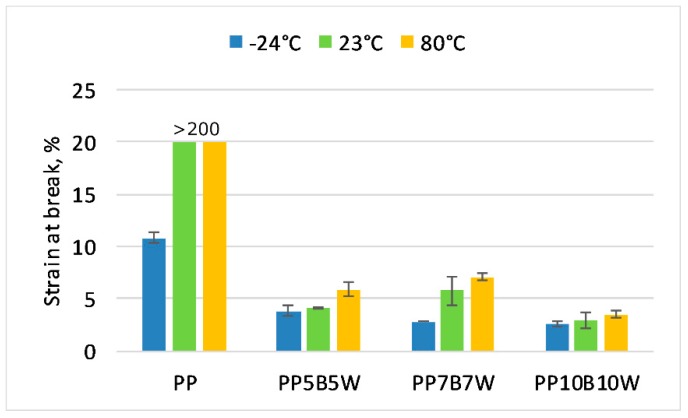
Strain at break of the tested materials at −24 °C, 23 °C, and 80 °C.

The influence of temperature on the change in the mechanical properties was determined. The results of the tensile and flexural test at −24 °C and 80 °C were compared with the properties measured at room temperature. The obtained results are presented in Figure 1, Figure 2 and Figure 3. It can be seen that 10 wt% addition of fibers resulted in an improvement in tensile strength by 38%, and successive additions of fibers had a less significant impact on the measured values. The tensile strength of neat PP determined at −24 °C increased by > 50% compared with that at room temperature and the increase in modulus of elasticity is was more than double. The glass transition temperature has impact on the mechanical properties of PP. The immobility of the macromolecules can be observed at very low temperature. It has been shown that modulus increases generally with an increase in fiber content. A significant increase of almost 2.5 times in modulus for 20 wt% total fibers was noted. At elevated temperatures, neat PP exhibited a 66% decrease in modulus of elasticity, while composites with 20 wt% fibers showed a reduction in modulus of elasticity by only approximately 35% compared to that determined at room temperature. The highest strain at break was observed for neat PP. The addition of fibers resulted in decrease in strain at break. The composites measured at 80 °C were characterized by higher strain at break than the composites measured at room temperature. Increasing progressive mobility of the restricted macromolecular zones can be observed if the polymer is heated. Due to the fact that certain molecular segments become more mobile at the transition temperatures, the material changes from a glassy hard state to a soft tough state [26]. 

The cellulose type of reinforcement is characterized by lower modulus of elasticity; thus, composites with wood fibers have lower stiffness than those with basalt reinforcement. From the comparison of the obtained results with literature data for PP composites with wood fibers [27] and basalt fibers without MAPP [28], it can be concluded that the composites described in this paper have more favorable mechanical properties. Composites with 20 wt% content of both fibers have 3 times more stiffness than composites with wood fibers and 2 times more stiffness than composites with basalt fibers without MAPP.

### 3.3. Flexural Properties

Table 3 summarizes the mechanical properties determined in the flexural test. The flexural modulus of elasticity and strength of tested composites increased with increasing fiber content. The strength for composites with 20 wt% fibers increased by 64%, whereas the increase in flexural modulus was triple. The obtained values were higher at −24 °C and lower at elevated temperatures; this was due to transition temperatures, as discussed for the tensile properties.

### 3.4. Rule of Hybrid Mixture and Halpin–Tsai equation

**Figure 4:**
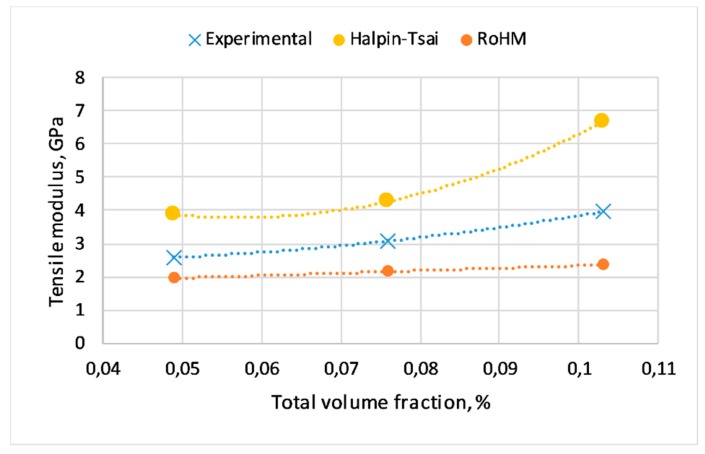
Comparison of modulus experimental results with RoHM and Halpin–Tsai equations.

The elastic modulus of composites was also predicted using RoHM and Halpin–Tsai equations. The experimental modulus values compared to the predicted values by making use of RoHM and Halpin–Tsai are presented in Figure 4. Because of the nature of the rule of mixture equation, a linear trend was observed for its predicted values. However, a nonlinear trend was observed for Halpin–Tsai equation. Figure 4 shows the difference in mean values between experimental and calculated elastic modulus values of each composite based on the volume fraction of the components. As shown, composites with higher volume fractions tend to have a higher difference between experimental and the calculated elastic modulus, whereas the lowest difference was observed for PP5B5W (consisting 5 wt% basalt fibers and 5 wt% wood fibers). It can be seen that the experimental values of modulus of elasticity obtained for the composites lie above the “mixtures rule” prediction. This indicates that the modulus exhibits a positive deviation from the mixture rule (i.e., the RoHM prediction).

### 3.5. Charpy Impact Test

**Figure 5:**
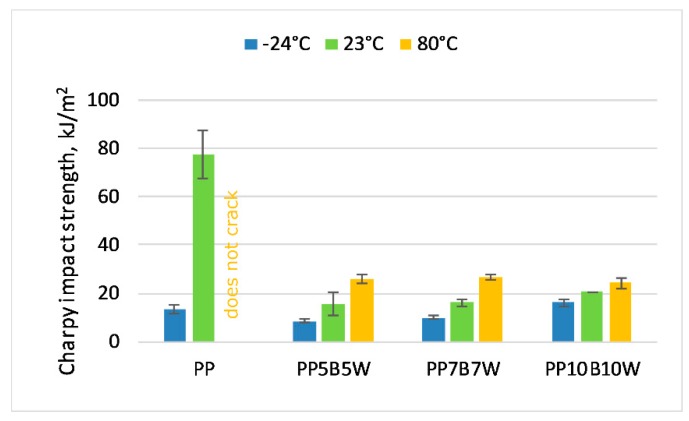
Unnotched Charpy impact strength of tested materials at −24 °C, 23 °C, and 80 °C.

The effect of fiber content on composite impact strength is shown in Figure 5. The composites samples prepared using the PP matrix showed a significant reduction in impact strength as the pure PP shows superior impact properties and the addition of fillers is known to decrease the impact properties of PP composites. The impact strength decreased after adding the fibers; however, it increased slightly as fiber content increased. The impact strength reduced by 80% for composites with 10 wt% total fibers. The decrease in impact strength at −24 °C was caused because of glass transition temperature of PP.

A heterogeneous system can be achieved by adding fibers to PP matrix. External load can lead to stress concentrations within these heterogeneities. Deformation and fracture behavior of composites is influenced by local stress maximums. The addition of fillers into the PP matrix leads to discrepancy in the overall process of crack propagation and fracture. The process begins with the plastic deformation of the matrix before the initial crack. The pull-out of fibers and debonding of particles affect the fracture toughness of composites [29].

### 3.6. Thermal Expansion

**Figure 6:**
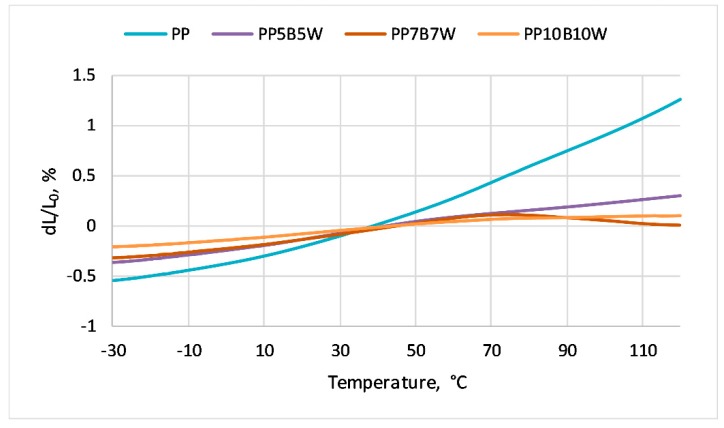
Thermal expansion curves of the tested composites.

The thermal curves of PP and its composites are presented in Figure 6. Temperature ranges presented in the graph refer to the linear nature of length changes, not to the full range of measurement. The values of linear coefficient of thermal expansion of the tested materials are presented in Table 4. Pure PP had the highest value. This is because thermoplastic polymers have high coefficients of linear thermal expansion due to weak secondary bonds between the chains. As the filler content added to PP increased, the linear coefficient of thermal expansion decreased by almost three times. This is a positive phenomenon due to the dimensional stability of elements in such composites. As discovered by Yang et al. [30], lignocellulosic filler is an appropriate material to prevent the thermal expansion of the composites caused by cold and warm atmospheric changes. The use of filler lowers the coefficient of thermal expansion. The coefficient of linear expansion, to a large extent, is dependent on the orientation and distribution of the reinforcing material.

### 3.7. Water Absorption

**Figure 7:**
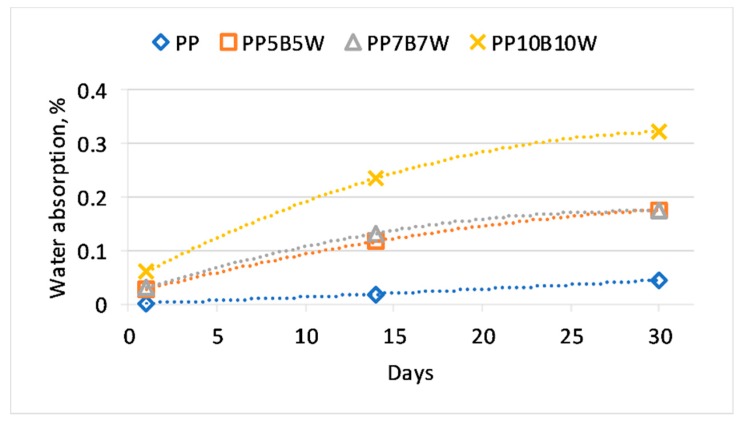
Water absorption of the tested materials.

Water absorption has a major impact on the behavior and stability of polymer composites, and it becomes more important when lignocellulosic fibers are added to the polymer matrix. In basalt/wood fiber hybrid composites, water absorption (Figure 7) increased with a growing filler content; however, the introduction of basalt fibers in hybrid composites provided an improved water absorption behavior compared to that of WPCs with equal amounts of filler. Specimens with 20 wt% wood flour absorbed slightly more than 1.2% moisture after 30 days of soaking in water [31].

**Figure 8:**
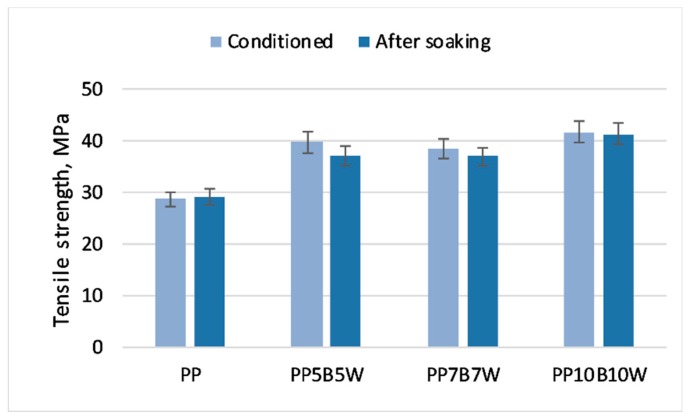
Tensile strength of the tested materials in the conditioned state and after soaking in water for 30 days.

**Figure 9:**
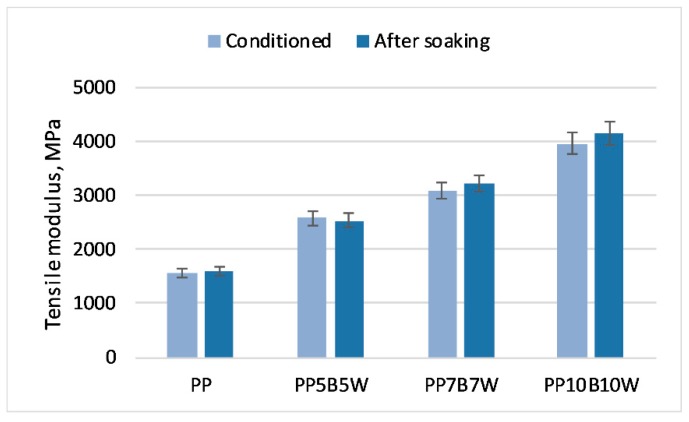
Tensile modulus of the tested materials in the conditioned state and after soaking in water for 30 days.

The changes in the tensile properties of the tested materials with changes in the amount of absorbed water are shown in Figure 8 and Figure 9. The expected decrease in strength parameters was moderate. Similar to the addition of 20 wt% carbon fibers to PP tested by Kada et al. [32], the addition of basalt fibers reduced water absorption compared to that of composites with wood fibers and stabilized strength properties in both tensile and flexural tests. The addition of the coupling agent MAPP also reduced water absorption because of the formation of covalent bonds of anhydride groups in MAPP with hydroxyl groups of cellulose; consequently, the polar functions of wood were no longer available for water molecules [33].

### 3.8. Fractographic Investigation

**Figure 10:**
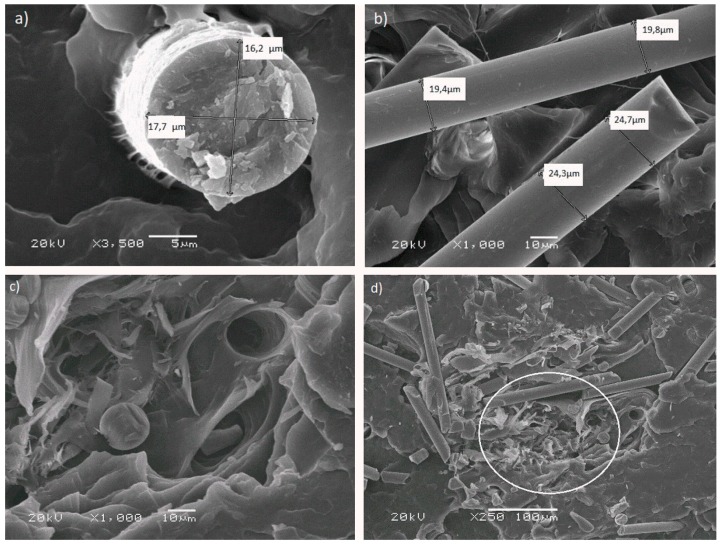
SEM images of the polypropylene (PP) composites with basalt and wood fibers (the fracture after the elongation test): (**a**) PP5B5W at 3500× magnification—a single basalt fiber in the PP matrix, (**b**) PP10B10W at 1000× magnification—a group of parallel basalt fibers, (**c**) PP5B5W at 1000× magnification—a broken basalt fiber and a “whole” fiber after pulling out one of them on the ductile fracture of PP, (**d**) PP5B5W at 250× magnification—area of entanglement of basalt and wood fibers.

SEM was performed to evaluate the state of dispersion and adhesion of basalt and wood fibers to the PP matrix. Micrographs of the fractured surfaces of tensile specimens are presented in Figure 10. After the elongation test, fractured specimens showed a combined characteristic from a brittle fracture (for higher addition of fibers) to a ductile fracture (for PP5B5W with a “pull-out” effect for parts of basalt fibers). Figure 10a and b show a single BF in the PP matrix. Various cross-sections of BFs were observed with the measured diameter of basalt fiber: 16–24 μm. Figure 10d shows the area of strong entanglement of wood and basalt fibers. Wood fiber has different morphology and surface conditions than those of artificial fibers (e.g., glass fiber and carbon fiber). This can be explained by the fact that wood fiber is a natural material, which means, that its microstructure contains many vessels (used for transportation of water and nitrite during the growth of the plant). Wood fibers consist of lignocellulose fibrils, and because of the addition of MAPP, they are uniformly embedded in the polymer matrix [34]. Functionalized PP (MAPP) creates a strong bond between the matrix and wood fibers. BFs has smooth surface, and fibers are not coated by the matrix. In Figure 10c it is easy to notice a hole on the fracture surface caused by fibers’ pull-out. This proves the need to achieve better adhesion between basalt fiber and matrix.

## 4. Conclusions

The present study allowed to determine the mechanical properties of the hybrid composites based on polypropylene. With regard to the mechanical properties, the addition of basalt fibers to WPCs increased stiffness, strength, and thermal resistance and decreased ductility and impact properties. A small 10 wt% addition of a mixture of wood and basalt fibers led to an approximately two-fold increase in elasticity modulus and a 30% increase in tensile strength. The incorporation of two types of fibers stabilized thermal resistance of composites. The linear coefficient of thermal expansion decreased by almost three times for composites with 20 wt% total fiber content. Moreover, the introduction of basalt fibers in hybrid composites provided improved water absorption behavior. The results indicate that hybridization of WPCs and basalt fibers can be successfully achieved. The obtained composites exemplify a formidable combination of properties for industrial applications.

## Figures and Tables

**Table 1:** Composition of tested materials. MAPP: maleic anhydride-grafted polypropylene.

Specimen	Matrix	Basalt Fiber Fraction, wt%	Wood Fiber Fraction, wt%	MAPP Fraction, wt%
PP	PP HP 500N	-	-	-
PP5B5W		5	5	3
PP7B7W		7.5	7.5	3
PP10B10W		10	10	3

**Table 2:** Basic physical and processing properties of the materials.

Index	Density, g/cm^3^		Shrinkage, %	Vicat Softening Temperature, °C
	Theoretical	Experimental		
PP	-	0.886	1.7	150.6
PP5B5W	0.961	0.947	0.5	154.3
PP7B7W	0.989	0.962	0.4	155.2
PP10B10W	1.017	1.001	0.3	157.3

**Table 3:** The influence of temperature on the change in the mechanical properties of the tested materials.

Index	Temperature, °C	Flexural Strength, MPa	Flexural Modulus, MPa
PP	−24	96.8 ± 1.2	4564 ± 120
	23	48.3 ± 0.9	1393 ± 90
	80	25.4 ± 0.6	561 ± 75
PP5B5W	−24	112.9 ± 1.4	5783 ± 115
	23	67.6 ± 1.2	2351 ± 85
	80	48.2 ± 0.6	1383 ± 95
PP7B7W	−24	103.5 ± 1.8	6256 ± 210
	23	69.8 ± 1.3	3179 ± 145
	80	43.6 ± 1.2	1444 ± 98
PP10B10W	−24	116.6 ± 1.5	7295 ± 245
	23	79.0 ± 1.1	4327 ± 165
	80	51.2 ± 1.7	2124 ± 110

**Table 4:** Linear coefficient of thermal expansion of tested materials.

Sample	Linear Coefficient of Thermal Expansion α × 10^−6^, 1/K
	−20 °C to 50 °C
PP	134.3
PP5B5W	73.8
PP7B7W	61.8
PP10B10W	51.9
